# Cellular Host Responses to Gliomas

**DOI:** 10.1371/journal.pone.0035150

**Published:** 2012-04-23

**Authors:** Joseph Najbauer, Peter C. Huszthy, Michael E. Barish, Elizabeth Garcia, Marianne Z. Metz, Sarah M. Myers, Margarita Gutova, Richard T. Frank, Hrvoje Miletic, Stephen E. Kendall, Carlotta A. Glackin, Rolf Bjerkvig, Karen S. Aboody

**Affiliations:** 1 Department of Neurosciences, City of Hope National Medical Center and Beckman Research Institute, Duarte, California, United States of America; 2 Division of Neurosurgery, City of Hope National Medical Center and Beckman Research Institute, Duarte, California, United States of America; 3 NorLux Neuro-Oncology, Department of Biomedicine, University of Bergen, Bergen, Norway; 4 NorLux Neuro-Oncology, Centre de Recherche Public de la Santé, Luxembourg, Luxembourg; Carl-Gustav Carus Technical University-Dresden, Germany

## Abstract

**Background:**

Glioblastoma multiforme (GBM) is the most aggressive type of malignant primary brain tumors in adults. Molecular and genetic analysis has advanced our understanding of glioma biology, however mapping the cellular composition of the tumor microenvironment is crucial for understanding the pathology of this dreaded brain cancer. In this study we identified major cell populations attracted by glioma using orthotopic rodent models of human glioma xenografts. Marker-specific, anatomical and morphological analyses revealed a robust influx of host cells into the main tumor bed and tumor satellites.

**Methodology/Principal Findings:**

Human glioma cell lines and glioma spheroid orthotopic implants were used in rodents. In both models, the xenografts recruited large numbers of host nestin-expressing cells, which formed a ‘network’ with glioma. The host nestin-expressing cells appeared to originate in the subventricular zone ipsilateral to the tumor, and were clearly distinguishable from pericytes that expressed smooth muscle actin. These distinct cell populations established close physical contact in a ‘pair-wise’ manner and migrated together to the deeper layers of tumor satellites and gave rise to tumor vasculature. The GBM biopsy xenografts displayed two different phenotypes: (a) low-generation tumors (first *in vivo* passage in rats) were highly invasive and non-angiogenic, and host nestin-positive cells that infiltrated into these tumors displayed astrocytic or elongated bipolar morphology; (b) high-generation xenografts (fifth passage) had pronounced cellularity, were angiogenic with ‘glomerulus-like’ microvascular proliferations that contained host nestin-positive cells. Stromal cell-derived factor-1 and its receptor CXCR4 were highly expressed in and around glioma xenografts, suggesting their role in glioma progression and invasion.

**Conclusions/Significance:**

Our data demonstrate a robust migration of nestin-expressing host cells to glioma, which together with pericytes give rise to tumor vasculature. Mapping the cellular composition of glioma microenvironment and deciphering the complex ‘crosstalk’ between tumor and host may ultimately aid the development of novel anti-glioma therapies.

## Introduction

Despite advances in surgical, radiation and chemotherapy treatments, patients with glioma have poor prognosis, with a median survival of 15 months and a 5-year survival rate less than 10% [1], [2], [3]. A more comprehensive understanding of glioma biology, including the role of the tumor microenvironment and tumor-host cellular crosstalk, is needed to develop more effective therapies for glioblastoma multiforme (GBM) [4].

In the adult brain, neural stem cells (NSCs) are a subpopulation of specialized astrocytes found in the subventricular zone (SVZ) of the lateral ventricles and the subgranular zone of the hippocampal dentate gyrus [5], [6], which have been implicated in learning, memory and in tissue regeneration [7], [8]. NSCs that originate in the SVZ of the rodent brain travel several millimeters in the rostral migratory stream to the olfactory bulb, where they differentiate into interneurons, which have been implicated in continuous replacement of neurons [9], [10]. NSCs in the subgranular layer of the hippocampus display a restricted migratory capacity and contribute to the genesis of dentate gyrus granule cells [10].

There has been longstanding interest in the medical and scientific community to develop NSCs for therapeutic purposes within the central nervous system, including NSC-mediated therapeutic gene delivery to malignant gliomas [11], [12], [13], [14], [15]. Although therapeutic studies are based on the transplantation of exogenous, genetically-modified NSC lines, one may envision that stimulating endogenous stem cells may serve as a means of antitumor therapy. Indeed, studies suggest that ‘naïve’, genetically-unmodified NSCs also have therapeutic effects [16] and that endogenous NSCs mobilized to gliomas have antitumor efficacy [17]. A greater understanding of the distribution, tropism and migratory routes of endogenous NSCs to gliomas will aid in the development of novel neurotherapeutics.

Previous studies of endogenous NSC homing to gliomas have mostly evaluated glioma cell line-based rodent tumors [17], [18]. The aim of our study was to investigate tumor-host interactions in animal models of orthotopic human glioma xenografts, with an emphasis on host-derived neural stem/progenitor cells (NSPCs) and endothelial progenitors [19] [20]. Our model allows to clearly distinguish between nestin-expressing cells derived from the human xenograft versus the host (mouse or rat)-derived nestin positive cells. Such distinction between these cell populations was made possible by using exclusively human-specific and exclusively mouse/rat-specific nestin antibodies, and double immunostaining methods that we have developed for use of primary antibodies when they are derived from the same species (e.g., mouse). We evaluated the contribution of endothelial progenitors to tumor angiogenesis. In addition to stem and progenitor cells, we investigated other cell populations that are known to be present in glioma and the tumor niche, such as astrocytes (identified by glial fibrillary acidic protein, GFAP) and pericytes that line the blood vessels (smooth muscle actin, SMA), and we assessed cell proliferation, migration and invasion. We determined the migration pattern, morphology and cell proliferation of host rodent neural precursors (identified by nestin expression) in response to implantation of three different human glioma cell lines (U251, U87, D566), as well as biopsy spheroids from GBM patients, which resulted in a panel of xenografts with differing growth patterns and invasiveness. We observed that host nestin-positive cells that originated in the SVZ ipsilateral to the tumor implant migrated towards the xenograft borders and deeper into the tumor regions. The observation that rodent nestin-positive cells were recruited to the parenchyma and neovasculature of glioma xenografts suggests a ‘crosstalk’ between the human tumor and rodent host compartments. Although the precise role of infiltrating NSPCs and endothelial progenitors in tumor progression is still poorly defined, we provide evidence for a putative role in tumor engraftment, invasion and angiogenesis.

## Results

### Host nestin-positive cells are attracted to glioma implants

The human glioma cell lines U251, U87 and D566 cell lines used in our current study were originally generated from malignant glioma patient material, and the take rates, immunogenicity and growth patterns of their lesions have been characterized previously [21], [22]. We found that all glioma cell-line based xenografts grew as hypercellular, pleomorphic and angiogenic with moderate (U251, D566) or little invasion (U87) into normal brain tissue. Although U251 tumors showed sharp, ‘pointy’ invasive protuberances, U87 tumors grew as large bulky tumors and displayed round, well-demarcated small satellites in the vicinity of the main tumor mass (occasionally we observed U87 small tumor satellites in the hemisphere contralateral to tumor implantation). Tumors from all three cell lines displayed some level of necrosis in the main tumor mass, but tumor satellites of U87-derived tumors were devoid of necrotic regions.

**Figure 1:**
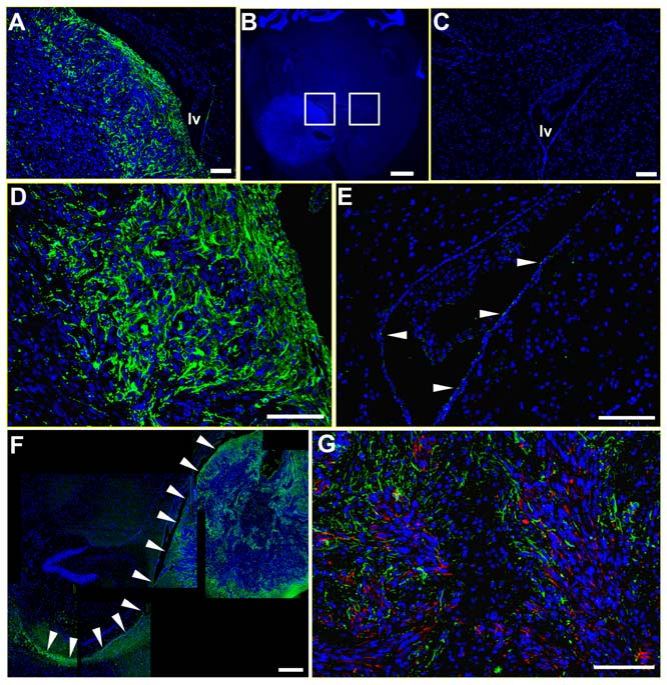
Host nestin-expressing cells are mobilized by human glioma xenografts in the mouse brain. Mouse nestin-positive cells (green) at the tumor border and peritumor brain tissue, as well as in deeper regions of the tumor mass (**A**, **D**) (boxed area on the left in **B** is shown enlarged in **A** and **D**). A mouse brain implanted with U251 human glioma cells (horizontal section; DAPI, blue) (**B**). Note the high cell density in the xenograft compared to the normal brain tissue. Higher magnification images of the lateral ventricle (hemisphere contralateral to the tumor) showing mouse nestin-expressing NSPCs (green, arrows) (**C**, **E**). Note the scarcity of nestin-positive cells and apparent lack of migration by these cells, suggesting little or no proliferation and migration by NSPCs from this region to the tumor. Recruitment of host nestin-positive cells (green) in the subventricular layer of the lateral ventricle ipsilateral to the tumor (**F**) (arrowheads indicate the wall of lateral ventricle). In this section, the lateral ventricle stretches from the hippocampal formation (caudal) toward the corpus callosum (rostral). Arrowheads show the apparent migration trajectory and pattern of NSPCs along fiber tracts of the deep cerebral white matter. Double-immunostaining for human (red) and mouse nestin (green) in the tumor, showing interdigitation of human and mouse cells (**G**). lv, lateral ventricle. *Bars* **A**, **C**, **D**, **E**, **G** 100 µm, **F** 500 µm, **B** 1 mm.

Mouse nestin-expressing cells were recruited to all three cell line xenografts, with a greater density of host cells observed at the tumor periphery in U251 xenografts (Fig. 1A). Composite images of DAPI-stained horizontal brain sections revealed the periventricular location of the xenograft in the right hemisphere, with boxes indicating magnified areas of interest in the ipsilateral and contralateral hemispheres (Fig. 1B, C). At high magnification, host nestin-positive cells were detected throughout the tumor, and a high density of such infiltrating cells was observed at the tumor periphery (Fig. 1D) and in deeper tumor regions, where clusters of host nestin-positive cells were also observed (Fig. 1F). Regions of tumor that appeared to be necrotic (depleted of DAPI stained cell nuclei) were surrounded by a halo of host nestin-positive cells (Fig. 1F), whereas the area lining the ventricle contralateral to the tumor had only few host nestin-positive cells (Fig. 1E). Host nestin-positive cells appeared to originate in the subventricular zone (SVZ) ipsilateral to the tumor implant, and were present in the ipsilateral SVZ at some distance (1–3 mm) from the tumor mass (Fig. 1F). Double-staining for human nestin (tumor) and mouse nestin (infiltrating host cells) revealed an interdigitation of host nestin-positive cells with human glioma cells in the tumor (Fig. 1G).

### Host nestin-positive cells migrate to the main tumor mass and small tumor satellites

**Figure 2:**
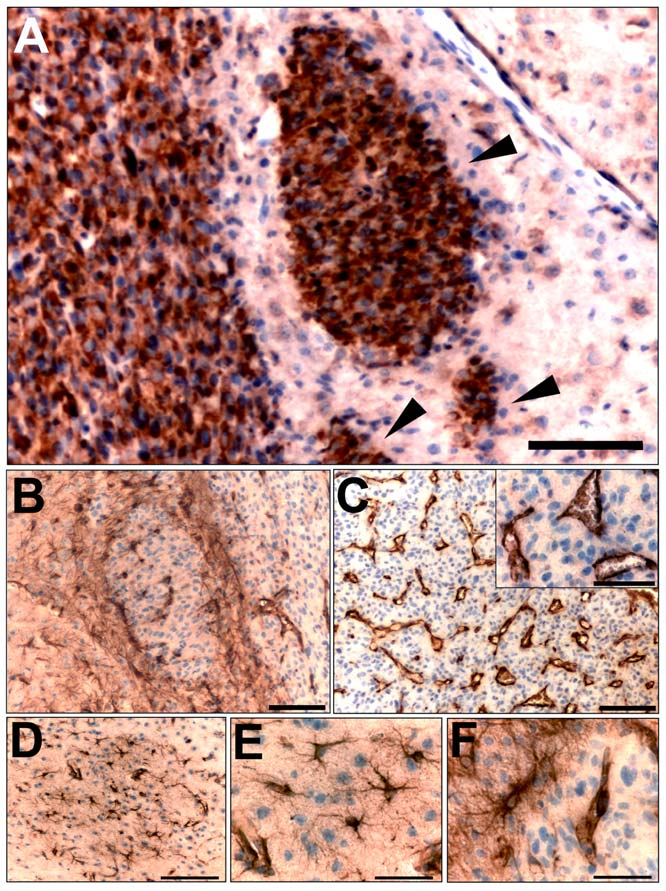
Mouse nestin expressing cells with different morphologies populate the primary tumor bed and the glioma small satellites. Immunostaining of U87 human glioma tumor bed and satellites) for human nestin (**A**) (arrowheads indicate tumor satellites). Host mouse nestin-expressing cells with rich arborizations encapsulate and infiltrate the tumor satellites observed in U87 xenografts (**B, D, E**). Mouse nestin-expressing cells form blood vessels in the main tumor mass (**C**; inset, higher magnification). Tumor satellite that was detected in the hemisphere contralateral to the main tumor mass (**D**). Mouse nestin positive cells displayed various morphologies, including rich arborizations (**E**) and spindle-shaped morphology (**F**). *Bars* **E**, **F** 50 µm, **A**, **B**, **C** 100 µm, **D** 150 µm.

In the U87 human glioma xenograft model, both the primary lesion and small tumor satellites stained uniformly for human nestin (Fig. 2A). Mouse nestin-positive cells encapsulated and infiltrated small tumor satellites both in the ipsilateral and contralateral side (Fig. 2B, D). Encapsulation of small tumor satellites by mouse nestin-positive cells was seen in satellites of sizes both smaller and larger than 100 µm, whereas infiltration by mouse nestin-positive cells was more pronounced in satellites that were >100 µm. Many of these mouse nestin-positive cells had rich arborizations, showing morphological similarity to astrocytes (Fig. 2B, D, E), while mouse nestin-positive infiltrating cells displayed a more elongated, spindle-shaped morphology (Fig. 2F). Blood microvessels in the tumor xenograft core stained intensely for mouse nestin (Fig. 2C), which suggests the contribution of host mouse nestin-positive cells to glioma angiogenesis. Nestin expression was seen in all tumor blood vessels covering the endothelial surface.

### Invasive and angiogenic GBM xenografts attract host nestin-positive cells

The biopsy xenograft model used in our study has been previously described and characterized [23], [24], [25]. We generated multicellular spheroids from glioma biopsies obtained from patients without passaging in monolayers. In contrast to glioma cell lines, the spheroids preserve the original tissue architecture of the human tumor, as well as other genotypic and phenotypic traits such as DNA ploidy and expression of tumor markers [26], [27], [28]. When intracranially transplanted into nude rats, the spheroids retain the same cellular phenotype, genotype and invasive growth as the patient tumor [23].

**Figure 3:**
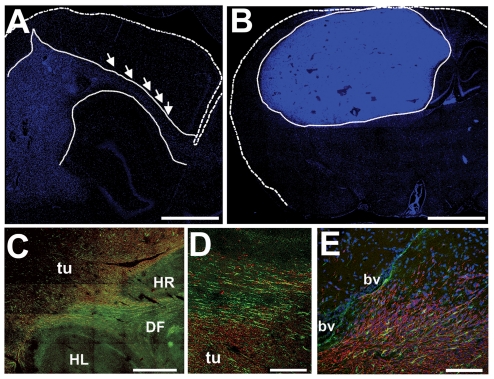
Peritumor infiltration of rat nestin-positive cells in human GBM biopsy xenografts. A low-generation xenograft lesion exhibiting invasive migration of tumor cells in the corpus callosum to the opposite hemisphere (**A**, arrows). Note also the infiltration of the cortex and hippocampus by invasive tumor cells (dashed lines, outline of cortical surface; dotted lines, outline of tumor border; blue, DAPI nuclear stain). An angiogenic high-generation GBM implant with high cell density and well-demarcated borders, dilated vessels and necrotic areas (areas devoid of DAPI (blue)-stained cell nuclei) (**B**) (dashed lines, outline of cortical surface; dotted lines, outline of tumor border; blue, DAPI nuclear stain). Rat nestin positive cells (green) accumulate around the human GBM xenograft border and infiltrate into the tumor (red, human nestin immunoreactivity) (**C**). A halo of host nestin-positive cells at the tumor border (**D**). Higher magnification of the xenograft lesion showing infiltration of elongated host nestin-positive cells into the tumor (**E**). *tu* tumor, *bv* blood vessel, *HL*, *HR* hippocampal formation left and right sides, *DF* dorsal fornix. *Bars* **E** 200 µm, **D** 500 µm, **C** 1 mm, **A**, **B** 2 mm.

We observed that the GBM biopsy spheroid xenografts in rats displayed two distinct phenotypes. When the xenografts were generated directly from patient material, the lesions were highly invasive, had low cell density and grew without angiogenesis. Glioma cells infiltrated the whole ipsilateral hemisphere and invaded the opposite hemisphere through the corpus callosum (Fig. 3A). However, xenografts generated from tumors that had been grown for several generations as serial *in vivo* passages in rat brains started to display dilated vessels, showed greater cell density, and exhibited only local invasion into brain tissue (Fig. 3B). Some of the high (fifth)-generation lesions included glomeruloid microvascular proliferations that recapitulated the morphological features observed in patients with GBM. Similar to what we observed in glioma cell-line implants in mice, glioma xenografts in rats displayed a halo of nestin-positive host cells that was localized to the tumor border and peritumor areas (Fig. 3C, D, E). Human nestin expression was uniform and robust in the tumor cells both in low- and high generation lesions (Fig. 3D, E). In addition, elongated host cells, with several processes, diffusely infiltrated the tumor edge and accumulated around brain vessels close to the xenograft (Fig. 3E). The elongated morphology of these infiltrating cells may suggest that they are moving towards their ‘destination’ within or adjacent to the tumor mass, especially to tumor blood vessels.

**Figure 4:**
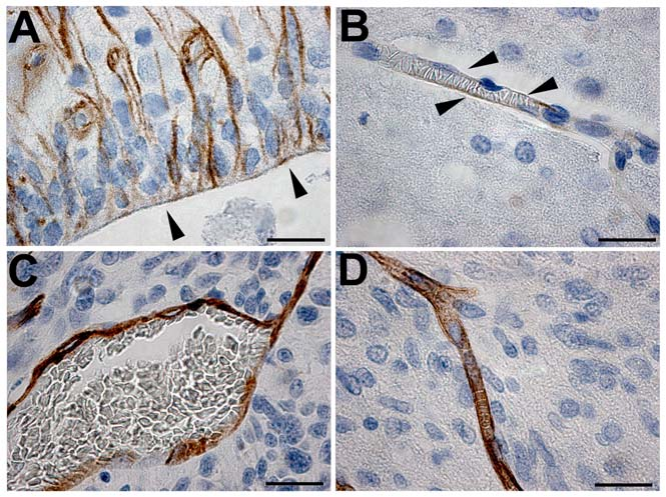
Expression of rat nestin in the host rat brain and tumor microvessels. Rodent nestin-positive cells (brown) in the wall of the third ventricle (arrowheads) (**A**). Weak, patchy expression of nestin was observed in some normal cortical microvessels. Arrowheads point to endothelial cells (**B**). Dilated tumor microvessel in a high-generation xenograft showing strong, relatively uniform host nestin expression (**C**). A tumor microvessel with normal, non-dilated morphology showing similarly strong staining for host nestin (**D**). *Bars* 20 µm.

We next investigated the expression of rodent nestin in the context of angiogenesis in rat brains bearing xenografts of GBM biopsy spheroids or that lacked such xenografts. In normal rat brains, nestin-expressing NSPCs were seen in the walls of the third ventricle (Fig. 4A) and the lateral ventricles, and the majority of cortical microvessels showed little or no nestin expression (Fig. 4B). However, in high-generation xenografts, staining for host nestin highlighted all tumor blood vessels (Fig. 4C) with a sharply defined endothelium-specific labeling pattern comparable to labeling with the endothelial marker, von Willebrand factor (data not shown). Markedly dilated vessels with irregular morphology were found close to the necrotic areas in the GBM xenografts. Rat nestin was similarly expressed in blood vessel walls in the immediate peritumor area and the tumor edge as well, although in these areas the microvessels assumed a normal (non-dilated) morphology. Strong nestin immunolabeling was also detected in normal-appearing, non-dilated microvessels in highly invasive lesions resembling gliomatosis cerebri in the low-generation, invasive GBM xenografts (Fig. 4D).

### Distribution and morphology of host nestin-positive cells in invasive and angiogenic glioma

**Figure 5:**
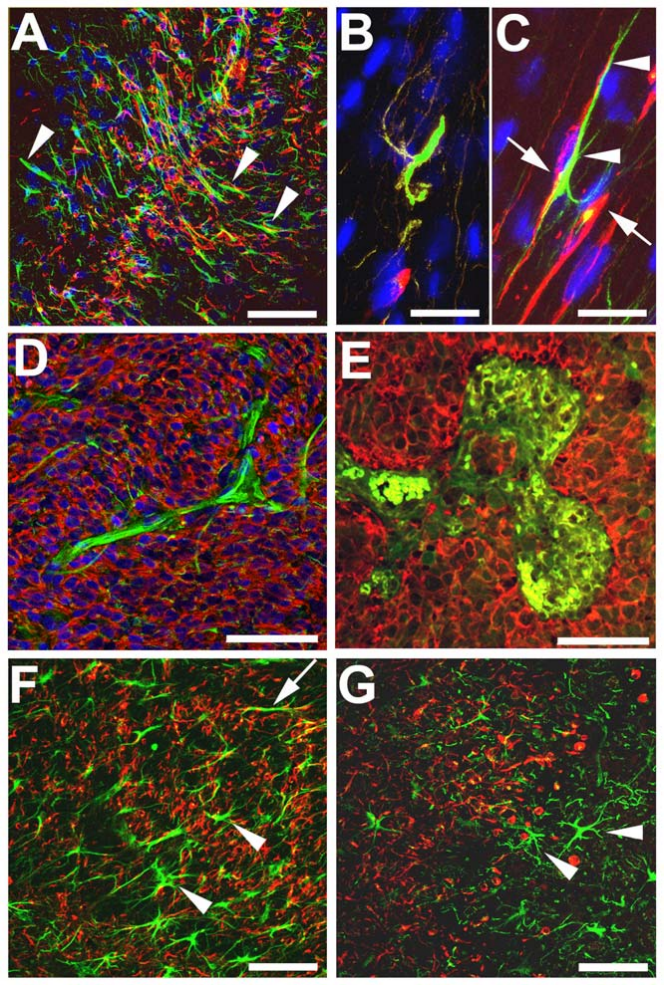
Morphology of rat nestin-positive cells and structures in invasive and angiogenic GBM biopsy xenografts. Elongated, bipolar rat nestin-positive cells (green, arrowheads) accumulate at the infiltrative edge of a biopsy xenograft (red) (**A**). Magnified view of cells in the tumor invasive zone with infiltrative single glioma cells [(arrowheads, rat nestin-positive cells (green); arrows, human nestin-positive glioma cells (red)] (**B**, **C**). A rat nestin-positive host cell (green) closely associated with an invasive human nestin-positive tumor cell (red, arrow), with the two elongated cell bodies juxtaposed to each other (arrowheads) (**C**). Rat nestin-positive vascular elements (green) in a high-generation human GBM xenograft (red) that induces angiogenesis (**D**). Host nestin-positive glomerular microvascular proliferation (green) typical of human GBM pathology recapitulated in the rat brain (**E**). Elongated rat nestin-expressing cells with terminal processes and star-shaped cells (green, arrowheads) in a GBM xenograft with locally invasive growth (red) and induction of angiogenesis (arrow) (**F**). Cells with astrocytic morphology stained for rat nestin (green, arrowheads) in a low-generation highly infiltrative xenograft with low tumor cell density (red) **G**). *Bars* **B**, **C** 10 µm, **F** 50 µm, **A**, **D**, **E**, **G**, **H** 100 µm.

In order to gain insight into the identity nestin-positive cells attracted by glioma, we studied the morphology of tumor-associated host cells after immunofluorescent double-labeling of human and rat nestin in tissue from the biopsy xenograft model. Along the invasive edge of the xenografts, we observed interdigitation of elongated, bipolar rat nestin-positive cells and tumor cells (Fig. 5A). Higher magnification revealed close physical contact between the elongated, spindle-shaped cells of the host and tumor cells with elongated morphology (Fig. 5B, C). We did not observe a co-localization of these two markers in the same cell, which would have indicated tumor-host cell fusion. However, we cannot exclude the possibility that such fusion events occurred, perhaps at very low frequency. Similar to the mouse xenograft models, rat nestin was expressed in tumor vessels and in glomeruloid microvascular proliferations in the biopsy xenografts (Fig. 5D, E). In diffusely infiltrative xenograft tumors, host nestin-stained cells were either elongated (invasive) cells with several terminal and central processes (Fig. 5F) or spread-out cells that resembled astrocytes (Fig. 5G).

### Host nestin-positive cells comprise a significant portion of the tumor mass

**Figure 6:**
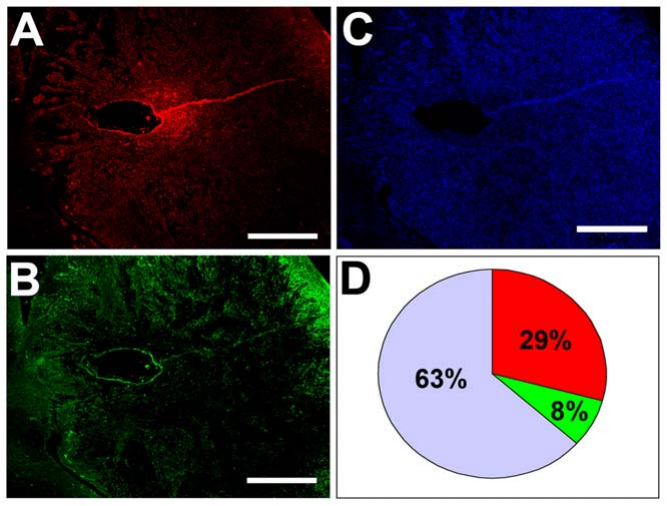
Quantification of areas with or without nestin expression in U251 glioma implants. Representative images of areas that contained cells expressing human nestin (red) (**A**) or host mouse nestin (green) (**B**), or stained with DAPI (blue) (**C**). Area measurements indicated that approximately 29% of the area contained cells that expressed human nestin (red), 8% contained cells that expressed mouse nestin (green), whereas 63% of the area was negative (blue-grey) for both human and mouse nestin (**D**). *Bars* **A**, **B**, **C** 300 µm.

Quantification of the tumor areas in sections of U251 glioma xenografts indicated that approximately 29% of the area contained cells that stained positive for human nestin (Fig. 6A) and 8% was positive for host mouse nestin (Fig. 6B), whereas 63% was negative for both human and mouse nestin (Fig. 6D). The areas that were devoid of human or mouse nestin-expressing cells, likely contained other cell types of the tumor stroma or extracellular matrix, which comprise a large portion of the tumor bed in gliomas. Some of the areas lacking human and mouse nestin expression included necrotic areas that were devoid of DAPI-stained cell nuclei (Fig. 6C).

### High cell proliferation is present within the tumor, at the tumor edge, and in the SVZ ipsilateral to the tumor

**Figure 7:**
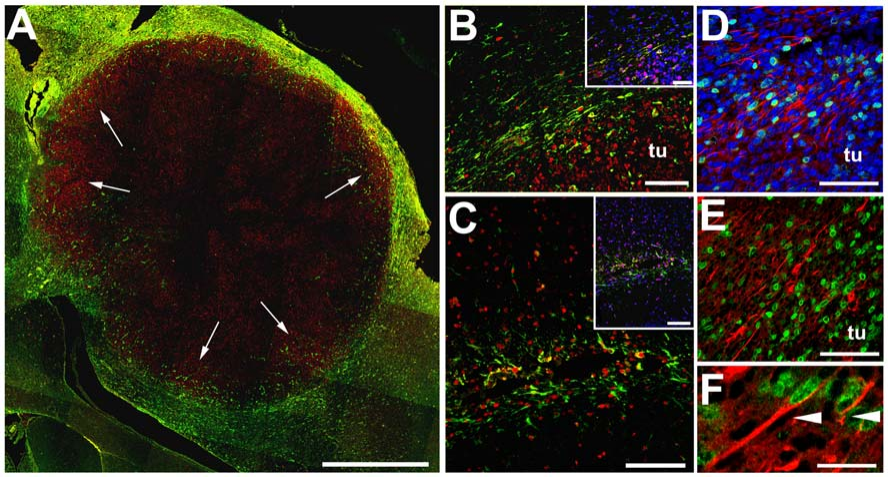
Cell proliferation at the tumor periphery in glioma xenografts. Confocal composite image of U251 human glioma xenografts in mice (**A**, **B**, **C**), and biopsy GBM spheroid xenografts in rats (**D**, **E**, **F**). Low magnification image shows a halo of host nestin positive cells (green) surrounding the tumor. Note the macroscopically demarcated border toward the brain tissue (**A**). Immunostaining of PCNA (red) at the tumor periphery and peritumor region indicates active proliferation in these areas (arrows). Higher magnification from panel **A** reveals that the majority of dividing cells are in the tumor, but some nestin-positive host cells in the peritumor brain tissue stain are also dividing (**B**). Extensive proliferation of host nestin-positive cells in the mouse subventricular zone ipsilateral to the tumor (**C**). Photomicrographs taken at the tumor periphery (lower right side of images, marked ‘tu’) and the surrounding brain tissue (upper left side of the images) reveal proliferating cells (Ki-67, green). Again, the majority of proliferating cells are in the tumor, but proliferating cells are also in the adjacent brain (**D**, **E**). Magnified image from **E** showing double-labeled dividing (Ki-67, green nuclei) rat nestin-positive cells (red cytoplasm) (**F**). *Bars* F 10 µm, **D**, **E**, **B** insert, **C** insert 50 µm, **B**, **C** 100 µm, **A** 1 mm.

We next used immunohistochemical double-labeling for mouse or rat nestin and proliferating cell nuclear antigen (PCNA) or Ki-67 to evaluate whether the infiltrative host cells were proliferating. Such investigation could reveal information regarding whether nestin-positive host cells proliferate at a distant site and then migrate to the tumor or whether a few of such host cells first migrate and then proliferate in the tumor context. In the mouse model, cell proliferation was most pronounced at tumor edges and focally around necrotic regions (Fig. 7A). At the tumor periphery, the majority of proliferating cells were tumor cells, whereas the brain parenchyma surrounding the tumor contained several host (mouse) nestin-positive cells that expressed PCNA, suggesting that these host cell were proliferating (Fig. 7B). The presence of a glioma implant induced proliferation of host nestin-positive cells in the ipsilateral, but not the contralateral SVZ (Fig. 7C). This may suggest that glioma and/or the brain tissue surrounding the tumor secrete soluble factors that may stimulate the proliferation of nestin-positive host cells. Similarly, cell proliferation was observed in the peritumor regions of biopsy xenografts in rats (Fig. 7D, E), with the majority of proliferating cells being glioma cells. Higher magnification revealed the presence of elongated bipolar cells that expressed both rat nestin and the proliferation marker Ki-67 (Fig. 7F).

### Some host nestin-positive cells display astrocyte morphology

**Figure 8:**
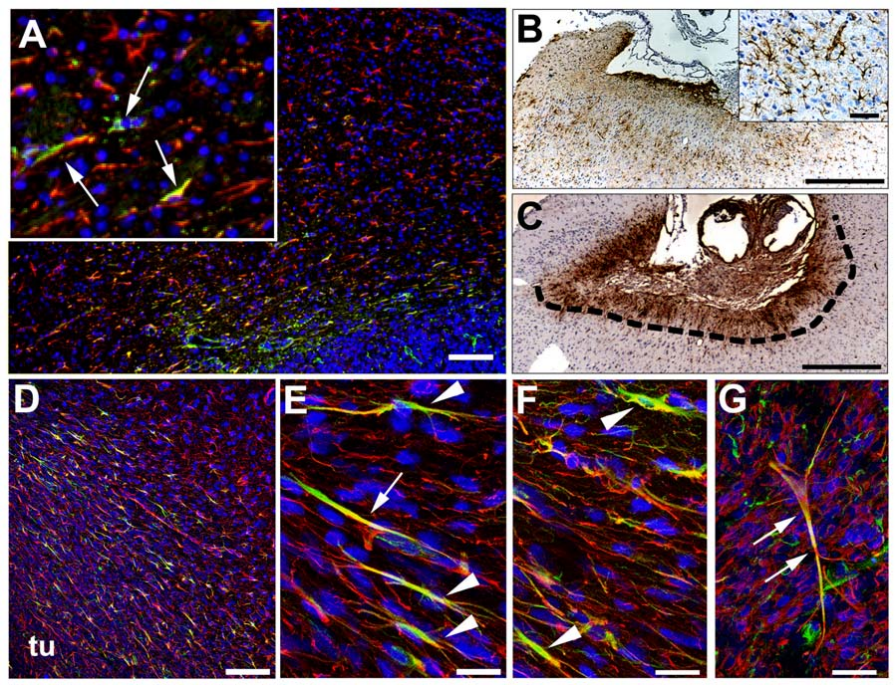
Localization of GFAP-expressing host cells and rodent nestin-positive cells in glioma xenografts. In mice, murine nestin-expressing host cells (green) at the U251 tumor border and also infiltrating into the tumor (**A**). These cells did not stain for GFAP, and were shown to be vascular elements. However, a few cells (yellow) expressed both mouse nestin and GFAP (**A**, inset, arrows). GFAP-expressing cells with characteristic branched morphology (astrocytes) were more numerous in the host brain tissue further away from the tumor border (**A**, red, GFAP; **B**, brown, GFAP). We also observed GFAP-positive cells with astrocyte morphology that extended astrocyte foot-like processes onto blood vessels in the vicinity of D566 tumor implant (**B** inset). Mouse nestin (brown) was strongly expressed at the immediate tumor border (dotted line) (**C**). GBM biopsy xenograft lesion showing a less demarcated front toward the brain tissue. Numerous double-immunostained cells were seen at the tumor border. GFAP strongly stained host astrocytes as well as glioma cells (**D**, red, GFAP; green, rat nestin). GFAP and rat nestin co-stained some host cells that showed diverse morphologies. Double-immunostained cells often had elongated cell bodies, with several terminal processes (**E**, **F**, red, GFAP; green, rat nestin). An elongated GFAP and rat nestin positive cell within the tumor center, with branched terminal processes (**G**). U251 (**A**), D566 (**B**, **C**), biopsy spheroid xenografts (**D**–**G**). *tu* tumor. *Bars* **E**, **F** 10 µm, **G** 15 µm, **A** insert 25 µm, **B** insert 75 µm, **A**, **D** 100 µm, **B**, **C** 500 µm.

Because neural stem cells have been proposed to be a subset of SVZ astrocytes, we labeled xenograft sections from tumors grown in mouse and rat brain for rodent nestin and glial fibrillary acidic protein (GFAP), a marker for astrocytes. In the tumor border zone, where mouse nestin-positive cells accumulated, elongated bipolar cells expressed both nestin and GFAP. Mouse nestin-positive blood vessels in the tumor were GFAP-negative (Fig. 8A, inset). We then compared adjacent tissue sections from tumor-bearing mouse brains stained for mouse nestin or GFAP. Large numbers of nestin-positive host cells were found at the immediate tumor border (Fig. 8C), whereas GFAP-positive host cells with typical astrocytic morphology were distributed loosely in a halo more distal to the zone containing mouse nestin-positive cells that surrounded the D566 human glioma implants (Fig. 8B). Some GFAP-positive cells were adjacent to and showed arbors projecting onto blood vessels (Fig. 8B, inset). In the GBM biopsy xenografts, similar peritumor accumulation of rat nestin positive cells was seen, with significant numbers of cells expressing both rat nestin and GFAP (Fig. 8D). In this case, both the xenograft as well as host astrocytes were positive for GFAP. Double-stained cells in the tumor periphery were often elongated with several terminal processes (Fig. 8E); however, plump cells with astrocytic morphology were also seen, possibly representing reactive astrocytes (Fig. 8F, top). Rat nestin and GFAP double-positive cells were more numerous in the tumor periphery, but were also found in the tumor core (Fig. 8G).

### Host nestin- and smooth muscle actin-positive cells: two distinct cell populations that contribute to tumor angiogenesis

**Figure 9:**
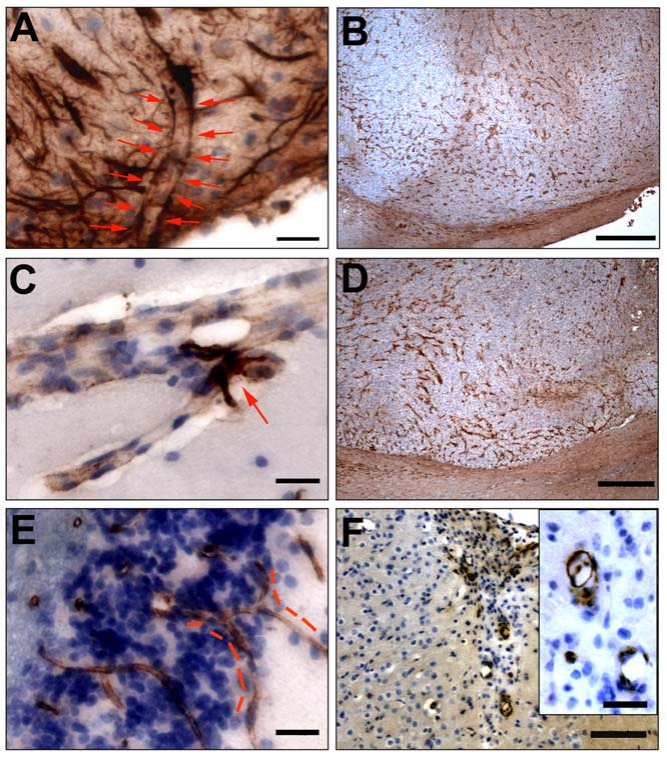
Participation of host nestin and smooth muscle actin-expressing cells in tumor angiogenesis. Blood vessel lined with mouse nestin-positive cells at the tumor periphery (**A**, arrows). Widespread vascularization with mouse nestin-positive cells in the tumor bed (**B**). Recruitment of mouse nestin-positive cells to a sprouting blood vessel branch (**C**, arrow). Smooth muscle actin-positive cells are also part of blood vessels in the brain tumor mass (**D**). Sprouting of mouse nestin-positive small blood vessels in the cerebellum of a glioma-bearing mouse (**E**, red dotted lines). Smooth muscle actin-positive cells at the invasive tumor edge (**F**) (inset, mouse nestin-positive blood vessel, blue, hematoxylin stain of cell nuclei). D566 xenograft (**A**, **C**, **E**), U87 xenograft (**B**, **D**). *Bars* **A**, **C**, **E** 25 µm, **F** 100 µm, **F** insert 25 µm, **B**, **D** 500 µm, **D** insert 50 µm.

**Figure 10:**
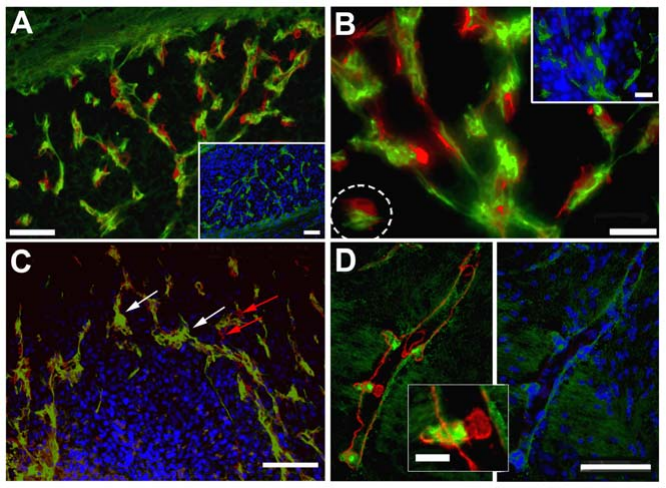
Host mouse nestin and smooth muscle actin-expressing cells are partners during glioma angiogenesis. Close contact was observed between host nestin (green) and SMA (red)-positive cells incorporated into blood vessels in a U87 xenograft 28 days post-implantation (**A**, higher power **B;** insets, nestin staining; dotted circle, putative ‘peg-socket’ structure). Cell nuclei are stained blue (DAPI). Onset of vascularization involving mouse nestin-(white arrows) and SMA-positive cells (red arrows) surrounding a small tumor satellite in A U87 xenograft 28 days post-implantation (**C**). Note the localization of mouse nestin- and SMA-positive cells at the periphery of this satellite, which descend in a pair-wise manner into the deeper parts of larger satellites (**A**, **B**). High-magnification image of a branching vessel in a D566 xenograft 10 days post-implantation showing SMA immunostained cells at the luminal lining and nestin expression at the sprouting front (**D**). U87 xenograft (**A**, **B**, **C**), D566 xenograft (**D**). *Bars* **D** insert 20 µm, **B** and **B** insert 25 µm, **A** and **A** insert 50 µm.

Given our observation that host nestin-positive cells contributed to the tumor neovasculature both in the mouse and in the rat xenograft models, we attempted to evaluate their spatial location in the vessels and mode of recruitment. Mouse nestin-positive cells were incorporated into vessels both in the periphery and core of U87 human glioma xenografts (Fig. 9A, B). Host cells with intense mouse nestin staining were also recruited into the site of vessel sprouting (Fig. 9C). Staining for smooth muscle actin (SMA), which is a marker for blood vessel-associated pericytes, exhibited a similar pattern as that for host nestin, highlighting microvessels in the main tumor mass (Fig. 9D), as well as in the tumor invasive edge (Fig. 9F). Mouse nestin also stained blood vessels in the cerebellum of tumor-bearing mice (Fig. 9E), although no tumor mass was apparent in the cerebellum. This may suggest that glioma in the cerebral frontal lobe may exert ‘long-distance’ effects, which may lead to upregulation of nestin expression in the cerebellar vasculature. SMA and nestin double-immunofluorescence labeling of U87 xenografts that were in the process of vascularization revealed that SMA-positive cells and nestin-positive cells were in close ‘pair-wise’ physical association with each other; however, they comprised two distinct populations of cells (Fig. 10A, B). Whereas SMA expression is expected to indicate pericytes/mural cells, nestin expression is thought to indicate endothelial progenitor cells. Both cell types were involved in the onset of vascularization in U87 small satellites (Fig. 10C). In addition, the leading edges of forming microvessels were capped by ‘sleeves’ of smooth muscle actin-positive cells, suggesting a role pericytes in the angiogenic process (Fig. 10D).

### SDF-1 and its receptor CXCR4 are expressed in the tumor and in the brain parenchyma surrounding the tumor

**Figure 11:**
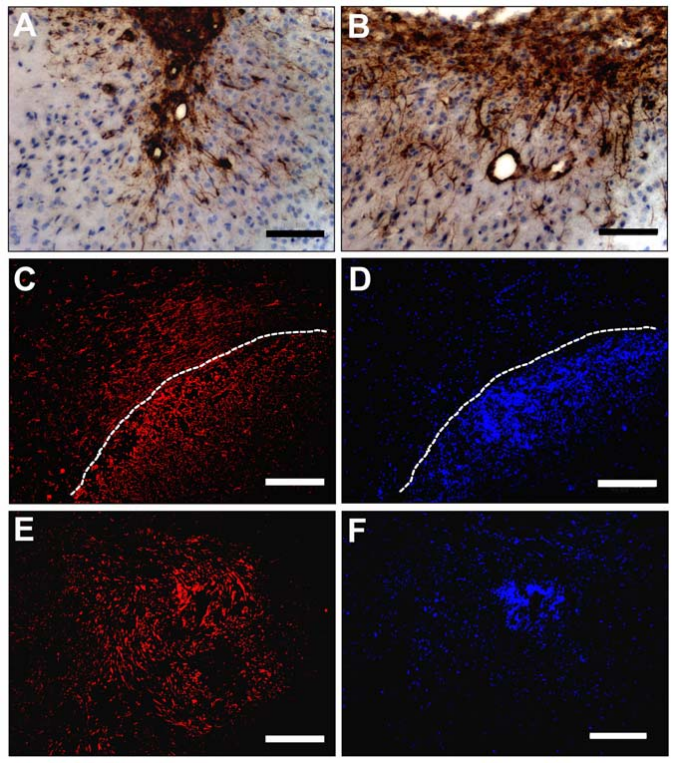
Expression pattern of SDF-1 and CXCR4 in glioma xenografts. SDF-1 (brown, A and B; red, C and E) is expressed at the tumor edge (**A**, **B**, **C**) and in the tumor center (**E**). SDF-1 staining was present in the peritumor blood vessels, where some of the signal appeared to be associated with the luminal surface of such blood vessels, indicating the presence of ‘SDF-1 trap’ in these vessels (**B**). SDF-1 expression (red) also appeared to extend beyond the tumor edge (**C**), where reactive astrocytes are known to express high levels of SDF-1. CXCR4 (blue) showed high expression at the tumor edge and in the tumor center (**D**, **F**). D566 xenograft (**A**, **B**), U251 xenograft (**C–F**). Dotted lines in **C**, **D** indicate tumor edge. *Bars* **A**, **B** 100 µm, **C–F** 200 µm.

Because SDF-1 and its receptor CXCR4 are involved in glioma invasion, we performed immunostaining for this ligand-receptor pair. The invasive edge, as well as the tumor periphery, was populated by SDF-1-expressing cells with elongated morphology and several terminal processes, reminiscent of invasive neural progenitors that were stained by host nestin (Fig. 11A, B). SDF-1 was also localized to peritumor blood vessels (Fig. 11B). Double-staining for SDF-1 and CXCR4 confirmed their expression in an increasing gradient toward the tumor periphery, as well as in a decreasing gradient away from the tumor in the peritumor host brain tissue (Fig. 11C, D). Both SDF-1 and CXCR4 were expressed in the tumor core, with CXCR4 being primarily localized around necrotic areas and more scattered elsewhere (Fig. 11E, F).

## Discussion

In this study we have evaluated the cellular host responses to orthotopic xenografts of glioma cell lines and GBM biopsy implants in rodent models. We observed that host stem/progenitor cells identified by rodent nestin expression displayed tropism for human glioma xenografts in both mice and rats, and that host cells originated and migrated from the ipsilateral SVZ to xenografts. We and others have previously reported specific tropism of exogenous and endogenous NSPCs to glioma xenografts [13], [16], [17]. During normal brain development, tissue regeneration and learning in rodents, NSCs originate in the SVZ and follow the rostral migratory stream to reach the olfactory bulb. This process is tightly regulated, and the cells do not disperse into the brain but rather follow a well-defined route [9]. In the pathological brain, the migratory patterns of NSCs change, with fewer new cells found in the olfactory bulbs and more cells found in the diseased cortex and corpus callosum [29]. NSCs were recruited from the SVZ ipsilateral to the tumor implant, while the contralateral NSC pools did not appear to be affected. In GBM biopsy xenografts in rats, the tumor border contained host nestin-positive cells with various morphologies: some showed a bipolar morphology characteristic of migrating cells with very thin elongated cell bodies, whereas others had several processes and assumed a star-like appearance characteristic of astrocytes. In low-generation invasive glioma lesions, nestin-expressing single cells with astrocytic or bipolar morphology were richly interwoven between infiltrating tumor cells. Previous investigations have shown that nestin-positive neural precursor cells accumulate at the site of mechanical brain injury, and by day 14 post-surgery, these cells display the branched morphology of reactive astrocytes [17]. On the other hand, NSCs around tumors displayed a bipolar morphology and persisted for at least 30 days, although in lower numbers, around the lesions. In comparison, our high generation biopsy spheroid-derived lesions grew in an obstructive way, creating a mass effect similar to the growth pattern of the glioma cell lines transplanted by Glass et al. (2005). Similarly, we generally observed a bipolar morphology in the nestin-positive host population of these lesions. In contrast, in low-generation xenografts, the tumor cells seamlessly infiltrated the brain, and we generally observed nestin-positive cells with an astrocytic morphology, comparable to what was seen at the site of mechanical injury (Glass et al., 2005). Possibly, in low-generation biopsy xenografts, after repair/regeneration of the injection site, the dispersed single glioma cells failed to stimulate further influx of neural progenitors from the SVZ. The previously recruited progenitors may have differentiated and displayed morphology similar to mature astrocytes.

The high (fifth) generation glioma implants were double-stained for GFAP and rat-specific nestin to evaluate the glial nature of the infiltrating host cells. GFAP stained both mature astrocytes and glioma cells. At the tumor border, the vast majority of nestin-positive cell bodies also expressed GFAP. These cells had the elongated cell bodies of invasive bipolar cells, although some also had several end-processes, resembling astrocytes. The bipolar cells may have been incoming neural progenitors, whereas those with branched morphology may have been reactive astrocytes. In the tumor, cells with elongated morphology, as well as cells with astrocytic morphology stained positively for both rat nestin and GFAP.

In both mice and rats, a host nestin-positive cell population (endothelial progenitors) contributes to human xenograft growth by assembling into the glioma microvasculature. In contrast, non-pathological brain microvessels showed little or no nestin expression. This confirms xenograft data from other tumor types, where nestin positive cells in tumor vessels have been identified as endotheliocytes [30]. In the high (fifth) generation GBM spheroid xenografts, we observed glomerulus-like microvascular proliferations, which stained intensely for host (rat) nestin. Such glomerulus-like vessels are a hallmark of GBM pathology [31], and likely represent non-functional vessels that might be responsible for generating the hypoxic environment in gliomas [32]. It is important to note that vascular endothelial growth factor (VEGF), when processed by matrix metalloprotesases (MMPs), causes generation of dilated, glomerulus-like vessels in tumors, whereas in the absence of MMPs more vascular sprouting is observed, which resembles the normal blood vasculature [33]. Because glioma pathology, invasion and angiogenesis are closely tied to the activity of various MMPs [34], and based on our current data, it is likely that recruitment of rat nestin-positive host cells by glioma reflects the intricate processes of glioma biology, including tumor hypoxia and angiogenesis.

In the mouse xenografts, staining for both host nestin and SMA showed similar staining pattern, illuminating the vascular network in the tumor bed. These two markers did not co-localize, but were expressed by two distinct, closely associated cell populations that together generated tumor microvessels. Based on their elongated morphology, luminal localization and relative abundance, host nestin-expressing cells are suggested to be endothelial cells or endothelial progenitors [19]. Indeed, the endothelial cell marker CD31 has been found co-expressed with nestin in the tumor vasculature of orthotopic xenografts of the pancreas in the nestin-GFP mice [30]. On the other hand, SMA is a marker of smooth muscle cells and mature pericytes, which may be derived from hematopoietic stem cells or bone marrow-derived pericyte progenitor cells that are recruited to the brain during glioma angiogenesis, suggesting a ‘long-distance’ effect in recruitment of cells that contribute to tumor angiogenesis [35], [36], [37], [38]. Furthermore, direct contacts between endothelial cells and smooth muscle cells have been described as ‘peg-socket’ contacts, where the basement membrane is absent. We detected structures resembling such contacts between SMA-positive cells and mouse nestin-positive cells (Fig. 10B). These cell-to-cell communication points have been described to contain tight, gap, and adherence junctions [39]. The contact between endothelial cells and pericytes leads to activation of latent TGF-β, which promotes pericyte differentiation via the ALK5 Smad2/3 signaling pathway [39]. Endothelial cells line the inside of blood vessels, whereas pericytes line the outside surface and interact with the basement membrane that surrounds the blood vessels [40]. In an analysis of the spatial localization of endothelial cells (CD31+) and pericytes (SMA+) during angiogenesis in spontaneous tumors of the pancreatic islet as well as in transplanted mammary and lung carcinomas, Morikawa et al found that, similar to our data, there was SMA-positive pericytic coverage or ‘sleeves’ at the leading edges of vascular sprouts [41]. In their study, pericytes in capillary microvessels formed by angiogenesis uniformly expressed SMA, in contrast to corresponding normal tissue, in which only arterioles and venules had SMA-positive adventitial cells. Our data show that in the brain, tumor capillaries were richly populated by SMA-positive accessory (pericytic/mural) cells, whereas the normal brain vasculature did not express SMA. In a recent study of 40 GBM patients Sica et al reported on the presence of nestin-positive, SMA-positive and CD105-positive cell populations associated with microvasculature in the peritumor area [42]. High microvascular density in the peritumor area correlated with a shorter survival time of the GBM patients, which suggests that the cell populations attracted by glioma may have an influence on the clinical progression of the disease.

The SDF-1/CXCR4 signaling axis has been shown to be a major player in the dissemination and metastasis of tumor cells, e.g., in breast cancer metastasis to the bone marrow [43], VEGF-induced neovascularization and retention of myeloid cells in normal and cancerous tissues [44], recruitment of vascular progenitors to glioma [38], and glioma invasion and growth [45], [46]. This chemokine/receptor system may be also involved in the homing of bone marrow-derived hematopoietic cells or neural stem or progenitor cells to tumors [47], [48]. In GBM, the expression levels of both SDF-1 and its receptor correlate positively with tumor grade, with CXCR4 being mainly localized to tumor endothelial cells, which likely use the SDF-1/CXCR4 axis to migrate during angiogenesis [49]. Exogenous NSCs that target human glioma xenografts in mouse brain localize to the tumor (including the hypoxic regions) and tumor edge, which displayed high levels of SDF-1 expression [50]. In normal brain, the expression pattern of SDF-1 and CXCR4 has been shown to coincide with that of NPCs, with virtually all nestin-positive cells in the SVZ also expressing CXCR4 [51]. Functional studies have confirmed the role of the SDF-1/CXCR4 system in NPC migration after neurological damage. For example, in cerebral stroke, the migration of NSCs to the injury site was attenuated by adding anti-SDF-1 blocking antibodies [52]. SDF-1 also promotes adult NPC proliferation as well as migration [53], [54], [55], and the SDF-1/CXCR4 signaling axis is involved in trafficking of normal stem cells as well as in metastasis of cancer cells [56]. We found that the location (peritumor halo, scattered single infiltrating cells in the tumor, and expression in tumor-associated blood vessels) and morphology (elongated single cells, cells with astrocytic morphology and small vessels) of SDF-1-expressing cells were identical to those of mouse nestin-expressing cells. This suggests that NPCs may express SDF-1, possibly indicating an autocrine stimulatory loop, or ‘trapping’ of SDF-1 by the recruited progenitors and vascular structures that arise from these cells.

*In vivo* migration of NSPCs is induced by vascular endothelial and astrocytic expression of SDF-1 [57]. Similarly, we observed SDF-1 expression in cells with astrocytic as well as endothelial morphology (Fig. 11), in addition to invasive cells that resembled infiltrating NPCs. CXCR4 expression localized to the same areas as that of SDF-1, but seemed to be less abundant both at the tumor-bordering brain and the tumor core. The exception was stronger expression of CXCR4 around necrotic foci as compared to SDF-1. Necrotic foci and the pseudopalisading regions are hypoxic and are the main sites of HIF-1α and VEGF expression in glioblastoma [58], [59], which may result in high expression of CXCR4. Indeed, over-expression of CXCR4 has been shown to be mediated by HIF-1 and VEGF [60], which is the main hypoxia-induced angiogenic pathway present in both vascularized cell line-based xenografts and in the high-generation lesions in the biopsy xenograft model [24].

Finally, the nestin-positive host cells recruited by gliomas may have effects other than those discussed above. For example, glioma cells generate considerable amounts of glutamate, which can lead to excitotoxity of neurons in the brain parenchyma surrounding the glioma mass, thus promoting the invasive migration and growth of glioma [61]. The presence of nestin-positive stem or progenitor cells in and around glioma may have protective effects against glutamate cytotoxicity. On the other hand, gliomas may co-opt some of the physiological properties of neural and mesenchymal stem cells for their advantage. Because neural and mesenchymal stem cells can release large amounts of TGF-β, a cytokine with immosuppressive potential, the anti-glioma immune response may be attenuated, resulting in tumor ‘escape’ from normal immunological surveillance [62], [63]. It should be noted that glioma cells may communicate with host cells in even more intricate ways; for instance, cell-cell fusion or horizontal gene transfer may result in exchange of genetic material between glioma and host cells [64], [65]. Furthermore, microvesicles (exosomes) that contain mRNA, microRNA and angiogenic proteins have been shown to be released by glioma cells, which can be taken up by normal host cells, such as brain microvascular endothelial cells [66]. A more recent and striking finding has been that glioblastoma stem-like cells can give rise to tumor endothelium [67], [68]. These data, together with our findings presented here, underscore the complex nature of malignant gliomas. Indeed, in addition to the cell types described in our study, the repertoire of cells attracted by or interacting with glioma is extremely diverse and includes neurons, oligodendrocytes, astocytes, microglia, cells of the immune system (B lymphocytes, various populations of T lymphocytes, macrophages), mast cells, and numerous others [69], [70], [71]. These cell types contribute to the microenvironment in which the tumor cells can proliferate, invade and destroy the normal brain parenchyma. However, some of these tumor-targeting host cells may serve also a protective function of the host by inhibiting tumor growth and invasion. It is hoped that further elucidation of the tumor and host interactions may aid the development of novel treatments for glioma, one of the most dreaded brain cancers.

## Materials and Methods

### Ethics statement

All patients gave a written informed consent for tumor biopsy collection and signed a declaration permitting the use of their biopsy specimens in scientific research. The study was approved by the Norwegian Regional Research Ethics Committee (http://helseforskning.etikkom.no/ikbViewer/page/komiteerogmoter/vest/medlemmer?p_dim=34986&lan=2&_ikbLanguageCode=n&region=10796). All protocols for the experiments involving rats, the handling of the animals and the surgical procedures were done in accordance with the Norwegian Animal Act and the local Ethics Committee approved the protocol. Mice were housed in a vivarium accredited by the American Association for Accreditation of Laboratory Animal Care. All protocols for mouse experiments were approved by the City of Hope Institutional Animal Care and Use Committee.

### Intracerebral implantation of glioma cell lines

The U251 and U87 human glioma cell lines were purchased from ATCC, the D566 cell line was a gift from Dr. Darell Bigner (Duke University, Durham, NC). Orthotopic tumor xenografts were generated by implanting these human glioma cell lines into the brains of 8-week-old female athymic *nude/nude* mice (Charles River). Animals were anesthetized (132 mg/kg ketamine and 8.8 mg/kg xylazine) and received stereotactically guided injections of 1−2×10^5^ tumor cells in sterile PBS (2 µL) through a 30-gauge Hamilton syringe over 3–5 min into the right forebrain (2 mm lateral, 0.5 mm anterior to bregma, tracked from a depth of 2.5 mm to 2.25 mm to 2.0 mm; 0.667 µL of cell suspension was injected at each depth) [13], [72]. The needle was retracted over 2–4 min, the burr hole occluded with bone-wax, Betadine applied to the surgical area, and the skin closed with skin glue or sutures. Analgesic (Buprenorphine, 0.05 mg/kg) was administered intra-peritoneally relieve post-operative pain. When mice appeared to be in discomfort or distress, as judged by independent animal care personnel with no knowledge of the protocol design, animals were euthanized.

### Intracerebral implantation of glioblastoma biopsy spheroids

Human GBM biopsy spheroids were prepared and maintained as described previously [26]. Briefly, patient biopsies obtained at surgery were transported to the tissue culture laboratory on ice. Thereafter, the tissue was minced in a laminar flow hood under sterile conditions using crossed scalpel blades. Tissue pieces (∼0.5–1 mm^3^) were incubated (37°C, 5% CO_2_) in flasks with agar overlay in Dulbecco's Modified Eagle's Medium supplemented with 10% fetal calf serum, 2 mM L-glutamine and non-essential amino acids. Tumors from nude rat brains were harvested and prepared similarly and were then implanted intracranially into another group of nude rats in order to grow the ‘higher generation’ tumors. Spheroids formed after a few days to one week in culture. Spheroid implantation into nude rats was performed as previously described [73]. Briefly, a burr hole was drilled 1 mm posterior to the bregma and 3 mm to the right of the midline suture, and 15 spheroids (diameter 400–600 µm) were injected into the corpus callosum region (at 2.5 mm depth) using a Hamilton type 7125 syringe (Hamilton, Bonaduz, Switzerland) before the wound was closed and the animals returned to their cages. The experimental protocol was approved by the Norwegian Animal Research Authority.

### Tissue processing

At varying time points after tumor cell implantation (U251, 76 days; U87, 28 days, D566, 10 days), mice were perfused intracardially with 4% paraformaldehyde (PFA) in PBS. The brains were harvested and post-fixed (4% PFA, 48 h, 4°C). For frozen sections, post-fixed brains were incubated (48 h, 4°C) in 30% (w/v) sucrose in PBS, embedded in optimal cutting temperature (OCT) compound and cryosectioned (10 µm thickness). Sections were thaw-mounted onto glass slides, air-dried and stored (−20°C) until further processing. For paraffin-embedded sections, post-fixed brains (without incubation in sucrose) were embedded in paraffin and sectioned (5 µm thick), and sections were mounted on glass slides and stored (room temperature) until further processing. Nude rats were intracardially perfused with sterile saline and the brains frozen in isopentane chilled by dry ice. Cryosections (10 µm) were mounted on glass slides and stored (−80°C) until further processing.

### Immunohistochemistry

Immunohistochemical staining (IHC) of cryosectioned or paraffin-embedded tissues was carried out as described previously [74]. Frozen sections were rehydrated in PBS, endogenous peroxidase activity was blocked (0.3% hydrogen peroxide in methanol, 20 min at room temperature) (for fluorescent IHC, no peroxidase blocking was performed), sections were blocked for non-specific antibody binding (blocking solution: 5% BSA, 3% normal serum from species in which secondary antibody was made, 0.1% Triton X-100 in PBS, 1 h at room temperature; or 5% BSA in PBS, 20 min). Sections were incubated (4°C, overnight) with primary antibodies in blocking solution, followed by several washes in PBS and reaction with biotinylated secondary antibodies (for some experiments, fluorophore-conjugated secondary antibodies were used). Immunostaining was visualized by chromogenic reaction (peroxidase/DAB) using the Vectastain Elite ABC kit (Vector Labs) or by reaction with fluorescently-labeled avidin.

Paraffin-embedded sections were deparaffinized, followed by antigen retrieval (10 mM citric acid, pH 6.0, and heating in microwave oven [2×5 min] or steam cooker, for all primary antibodies, except for SDF-1 and smooth muscle actin for which 1 mM EDTA, pH, 8.0, and heat treatment was used). Sections were then processed for peroxidase/DAB or fluorescent IHC. For fluorescent double-labeling IHC, primary antibodies generated in different species were used. For fluorescent double-labeling with primary antibodies from the same species, we used a modified IHC method using an avidin-biotin blocking kit and blocking with non-biotinylated secondary antibodies. Primary antibodies were omitted, and secondary antibodies were included during staining of tissue sections that served as negative controls.

Primary antibodies used were: CXCR4 (rabbit polyclonal, 5 µg/mL final concentration, Abcam, ab2074), GFAP (rabbit polyclonal, 75 µg/mL, Sigma, G9269; chicken polyclonal, 1∶1000 dilution, Chemicon, AB5541), human nestin (rabbit polyclonal, 1∶200, Chemicon, AB5922; mouse monoclonal, 5-10 µg/mL, Chemicon, MAB5326), Ki-67 (rabbit polyclonal, 1∶200, Chemicon, AB9260; rat monoclonal, 73 µg/mL Dako, M7249), mouse/rat nestin (mouse monoclonal, 5–10 µg/mL, Chemicon, MAB353), PCNA (mouse monoclonal, 10 µg/mL, Chemicon, MAB424), SDF-1 (mouse monoclonal, 5 µg/mL, R&D Systems, MAB350), smooth muscle actin (mouse monoclonal, 0.7 µg/mL, Dako, M0851).

Secondary antibodies used were: anti-mouse IgG (biotinylated, 2 µg/mL, Vector Labs, BA-2001), goat-anti-mouse IgG_1_ (FITC-conjugated, 5 µg/mL, Southern Biotech, 1070-02), anti-mouse IgG (non-biotinylated, 5 µg/mL, Vector Labs, AI-2001), anti-rabbit IgG (biotinylated, 2 µg/mL, Vector Labs, BA-1000), goat-anti-rabbit IgG (Texas Red conjugated, 5 µg/mL, Southern Biotech, 4010-07) anti-rat IgG (biotinylated, 2 µg/mL, VectorLabs, BA-4001), goat-anti chicken biotinylated (7.5 µg/mL, Vector Labs, BA-9010).

Chromophores and other reagents used for immunohistochemistry: Avidin-AMCA (1 µg/mL, Vector Labs, A-2008), ExtrAvidin-Cy3 (1∶200, Sigma, E4142), Avidin-FITC (1 µg/mL, Vector Labs, A-2011), Avidin-Texas Red (1 µg/mL, Vector Labs, A-2016), Avidin-biotin blocking kit (Vector Labs, SP-2001), bovine serum albumin (Sigma, A7906), citric acid (Sigma, C8532), Cytoseal 60 (VWR, 48212-187), DAB (3,3′-diaminobenzidine) substrate kit (Vector Labs, SK-4100), fluorescent mounting medium (Dako, S3023), DAPI (1 µg/mL, Sigma, D-9542), Hematoxylin (Sigma, HHS32), normal horse serum (Vector Labs, S-2000), normal rabbit serum (Vector Labs, S-5000), PBS (phosphate buffered saline, pH 7.4), Triton X-100 (Sigma, T9284), Vectastain Elite ABC kit (Vector Labs, PK-6100).

### Image processing

Low- and high-magnification images were obtained using a Nikon Eclipse TE2000-U microscope (Nikon Instruments) equipped with brightfield and fluorescence illumination. Images were recorded and stored using SPOT Advanced and Adobe Photoshop software. Some images were obtained by confocal microscopy using a Zeiss LSM 510 confocal microscope (Carl Zeiss Microimaging Inc.), and multi-panel composite images were constructed using the MetaMorph software. NIH Image J software was used for area measurements of tissues stained for human nestin and mouse nestin.
